# Step-by-step laparoscopic transcystic common bile duct exploration in choledocholithiasis: technical tips, special scenarios, lessons and results from over 100 cases

**DOI:** 10.1007/s00464-026-12736-2

**Published:** 2026-04-13

**Authors:** Víctor Nieto Barros, Laura Alonso Murillo, Raúl Castañeda-Vozmediano, Carlos González del Arco, M. Victoria Vieiro Medina, Carlos García Vásquez, Santos Jiménez-Galanes Marchán

**Affiliations:** 1 School of Medicine, Universidad Francisco de Vitoria (UFV), Madrid, Spain; 2 Department of General Surgery, Infanta Elena University Hospital, Av. de los Reyes Católicos, 21, Valdemoro, 28342 Madrid, Spain

**Keywords:** Transcystic, Choledocholithiasis, Laparoscopic, Common bile duct exploration, Stet-by-step

## Abstract

**Background:**

Laparoscopic transcystic bile duct exploration has progressively evolved as an effective single-stage approach for the management of choledocholithiasis in patients with gallbladder in situ. Although endoscopic retrograde cholangiopancreatography followed by cholecystectomy remains widely practiced, growing evidence supports the transcystic approach as a safe, minimally invasive, and cost-effective alternative.

**Methods:**

We present a standardized, step-by-step description of the laparoscopic transcystic technique developed through our experience in over 100 consecutive patients. The procedure is detailed, including the management of specific intraoperative scenarios. Technical tips and tricks are provided to optimize performance in cases of narrow cystic ducts, impacted or large stones, prior biliary prostheses and complex inflammatory conditions. In addition, we developed a learning-curve modeling framework to quantify how surgical experience influences key outcomes and to estimate the procedural volume required to achieve reproducible and acceptable results in other centers adopting this technique.

**Results:**

The standardized technique proved feasible and reproducible across a broad range of clinical presentations. The transcystic approach allowed successful clearance of the bile duct in the vast majority of patients. Our cumulative experience demonstrates that the technique can be safely adopted regardless of cystic duct diameter, number or size of stones, or patient characteristics.

**Conclusion:**

The transcystic approach represents a valid, safe, and reproducible first-line technique for choledocholithiasis in patients with gallbladder in situ. Its standardization facilitates consistent outcomes and significantly shortens the learning curve, enabling its adoption as a routine practice in centers experienced in advanced laparoscopic biliary surgery.

**Supplementary Information:**

The online version contains supplementary material available at 10.1007/s00464-026-12736-2.

The development of endoscopic techniques has led in recent years to a progressive change in the management of various clinical situations encountered by surgeons. Cholelithiasis is one of the most frequent digestive pathologies, accounting for up to one third of the workload in any general surgery department [1]. Within this condition, choledocholithiasis is estimated to affect approximately 5–10% of patients with cholelithiasis, although this figure may be underestimated due to the dynamic nature of the disease [1].

Traditionally, patients with choledocholithiasis and gallbladder in situ were treated with endoscopic retrograde cholangiopancreatography (ERCP) followed by cholecystectomy. However, the evolution of laparoscopic bile duct exploration (LBDE) has progressively changed this paradigm. There is growing evidence supporting that the single-stage approach is both effective and safe compared with the conventional two-stage treatment and has the potential to reduce both length of hospital stay and associated costs; however, its implementation is contingent upon surgical expertise and the availability of necessary resources [2–5]. Nevertheless, the potential benefits of this strategy may still be underestimated, as some studies do not differentiate between the transcholedochal and transcystic approaches, overlooking the specific advantages of the second one. The transcystic approach, performed using choledocoscopes with diameters of 2.8–3.5 mm, has shown superior outcomes compared with the transcholedochal technique, including lower rates of bile leakage, reduced operative time, shorter hospital stay, and lower overall costs [2, 5–12].

Consequently, we advocate transcystic common bile duct exploration (tLCBDE) exploration as the primary approach for managing patients with choledocholithiasis and gallbladder in situ, irrespective of cystic duct diameter, stone burden, or patient characteristics as noted also in recent contemporary data where tLCBDE was evaluated as first-line treatment in a large unselected cohorte [13, 14].

Concerns regarding technical complexity and the learning curve remain important barriers to widespread adoption; therefore, understanding the impact of surgical experience is essential to assess the safety, reproducibility, and applicability of this technique.

In this article, we describe a standardized step-by-step technique refined through our experience in over 100 consecutive cases, as well as a series of technical tips and tricks for particular scenarios we have encountered during clinical practice. Given the limited availability of robotic surgery and the growing body of evidence supporting tLCBDE, studies such as this one gain additional relevance by promoting the dissemination of techniques that can be safely and effectively implemented in different surgical contexts.

## Materials and methods

Since June 2020, we have prospectively recorded the experience of 100 patients who underwent elective tLCBDE for confirmed or suspected choledocholithiasis. The diagnosis was established using ultrasound (US), computed tomography (CT), magnetic resonance cholangiopancreatography (MRCP), or prior ERCP. Patients with analytical findings suggestive were also included. No extensive patient selection was applied. This strategy is consistent with recent evidence demonstrating the feasibility of tLCBDE in the majority of patients with choledocolithiasis [13]. The exclusion criteria included patients with neoplasms of the biliary tract and those initially managed with non-transcystic approaches (such as choledochotomy or transinfundibular techniques). All patients were followed for a minimum of 6 months, with clinical and laboratory assessments performed at 1 and 6 months and imaging (predominantly MRCP) at 6 months. By elective we refer to non-emergent surgery. This procedure requires specialized equipment and trained personnel not available 24/7 in our institution; therefore, patients were not operated on immediately but after a mean delay of 2.25 days, once the necessary resources were organized.

The interventions were performed predominantly by the same surgical team—most notably by the same lead surgeon. Over time, progressive modifications were introduced until achieving the complete standardization of the technique described below.

### Statistical analysis

Variables were summarized using measures of central tendency and dispersion for continuous data and frequencies and percentages for categorical variables. Missing data rates were reported and handled using machine-learning–based imputation. Postoperative outcomes were described according to the cumulative number of procedures performed.

To model the surgical learning curve, univariate and multivariable generalized additive models (GAMs) were applied, adjusting for age, preoperative leukocyte count, bile duct diameter, stone size, and age-adjusted Charlson Comorbidity Index (CCI). The association between procedural volume and seven intra- and postoperative outcomes (length of stay, operative time, pancreatitis, overall complications, bile leak, biliary stricture, and cholangitis) was assessed. Penalized spline functions were used to capture potential non-linear relationships while limiting overfitting. For each model, effect estimates, statistical significance, and explained variance were reported, and learning curves were visualized. When procedural volume predicted meaningful improvement in outcomes, the estimated number of cases required to achieve satisfactory results was calculated. Statistical significance was set at *p* < 0.05. Analyses were performed using R software.

## Results

A total of 100 patients were included. The mean age was 62.61 ± 16.72 years (median 62.08; interquartile range [IQR] 52.43–76.67; range 21.24–89.92). Sixty-one patients were female (61%) and 39 were male (39%). According to the ASA physical status classification, 21 patients were ASA I (21%), 43 ASA II (43%), 29 ASA III (29%), 5 ASA IV (5%), and 1 patient had unknown status (1%).

Preoperative laboratory tests obtained 24–48 h before surgery showed a mean total bilirubin of 2.34 ± 2.77 mg/dL (median 1.20; IQR 0.52–3.00; range 0.20–14.06), gamma-glutamyl transferase (GGT) of 457.21 ± 557.82 U/L (median 348.00; IQR 99.00–609.00; range 11.00–4418.00), and alkaline phosphatase (ALP) of 247.69 ± 250.47 U/L (median 169.00; IQR 104.00–334.00; range 10.00–2022.00).

Preoperative imaging demonstrated a common bile duct diameter of 9.00 ± 3.58 mm (median 8.00; IQR 7.00–11.00; range 3.00–20.00) and a mean choledocholithiasis size of 6.48 ± 4.31 mm (median 6.00; IQR 3.50–8.00; range 0.00–20.00). Regarding the number of bile duct stones, 46 patients presented one stone (48.42%), 12 had two (12.63%), 9 had three (9.47%), and 9 had four or more stones (9.47%). Debris without defined calculi was observed in 9 patients (9.47%), a clear common bile duct in 8 (8.42%), and other findings (tumoral dilatation or dilatation without intraoperative lithiasis) in 7 patients.

Intraoperative cholecystitis was present in 47 cases (50%), absent in 47 (50%), and unknown in 6 patients.

Lithotripsy was performed in 54 patients (54%). When used, the mean energy applied was 0.97 ± 0.44 J (median 1.20; IQR 0.50–1.20; range 0.50–1.80) and the mean frequency was 6.20 ± 1.43 Hz (median 5.00; IQR 5.00–8.00; range 5.00–8.00).

The working instruments employed were NGage® Stone Extractor in 39 cases (39%), Dormia® NStone® in 13 (13%), SpyBite™ biopsy forceps in 8 (8%), Fogarty catheter in 3 (3%), other devices (Elefant®, combined Dormia® and NGage®, or pediatric nasogastric tube) in 3 (3%), and no additional instrument in 34 procedures (34%).

The mean operative time was 189.74 ± 91.20 min (median 183.00; IQR 125.00–265.00; range 35.00–385.00).

The procedure is performed under general anesthesia with the patient in the supine position, legs apart, and slightly in the anti-Trendelenburg position. Pneumoperitoneum is established using a Veress needle inserted into the left hypochondrium (Palmer’s point). A 12 mmHg pneumoperitoneum was maintained using a valveless insufflation system (AirSeal®, CONMED, Utica, NY, USA). Subsequently, a laparoscopic approach with three trocars is employed. The first trocar (11 mm, optical) is inserted in a supraumbilical position for introduction of the laparoscope. After inspection of the abdominal cavity and removal of the Veress needle, the remaining two trocars are placed for the surgeon’s working hands. The AirSeal® trocar is typically positioned for use with the surgeon’s right hand.

1. The procedure begins with an anterograde cholecystectomy (from the infundibulum to the fundus), completely freeing the gallbladder from its bed while preserving its attachment to the cystic duct. Ultrasonic dissection and sealing of the cystic artery are performed using the Harmonic® device (Ethicon Endo-Surgery Inc., Cincinnati, OH, USA). Using this device, cholecystectomy can be safely performed with three trocars in most cases; however, in complex cholecystectomies, placement of a fourth trocar was required. Complete dissection of the junction between the cystic duct and the common bile duct is crucial to achieve adequate mobilization and facilitate subsequent exploration. Once this step is completed, an Endoloop® (Ethicon, Johnson and Johnson, Cincinnati, OH, USA) is placed at the junction of the infundibulum and cystic duct and externalized through the right hypochondrium with the assistance of an EndoClose**®** device (Covidien, Medtronic, Mansfield, MA, USA). External traction is then applied to the gallbladder to create a 90° angle between the cystic duct and the common bile duct, as illustrated in Fig. 1. A Crile-type hemostatic clamp is positioned at the skin exit point of the Endoloop to maintain external traction which is both adynamic and constant, allowing the surgeon to work hands-free and facilitating a clearer surgical field.

**Fig. 1:**
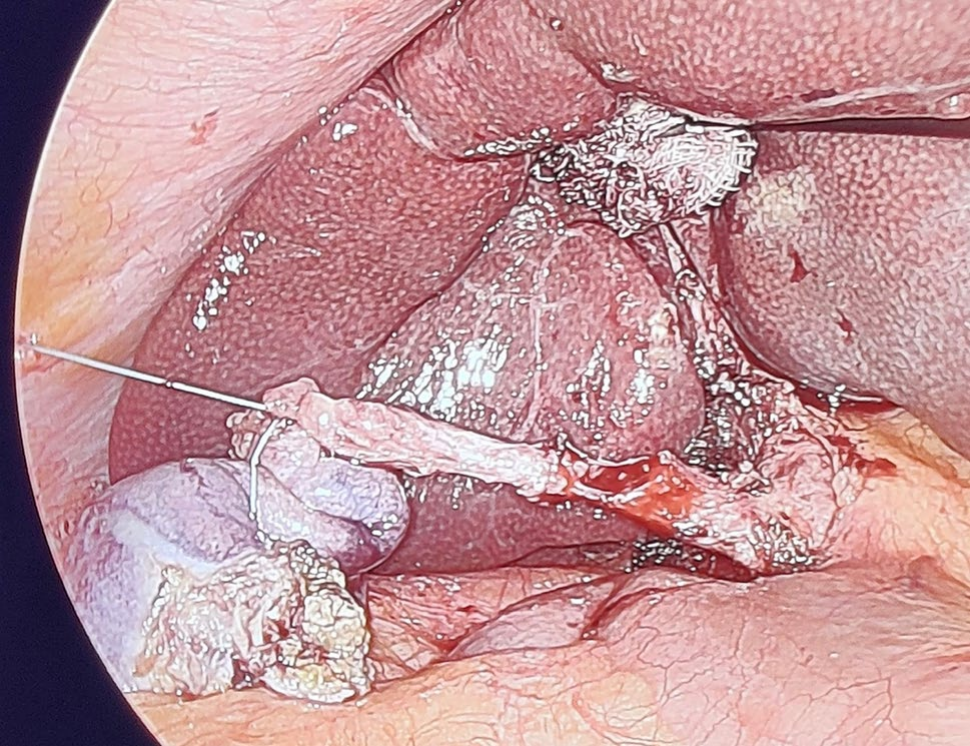
External traction with an Endoloop® applied at the junction between the infundibulum and the cystic duct to create a 90° angle with the common bile duct. This configuration facilitates subsequent transcystic exploration and choledochoscope alignment
2. A partial transverse incision was made on the anterior wall of the cystic duct, approximately 0.5–1 cm from its junction with the hepatic duct. Following this incision, the cystic duct was dilated with the assistance of a right-angle dissector. At this stage, if not previously placed, a fourth trocar was introduced and positioned to facilitate choledochoscope insertion, ensuring alignment with the line of traction on the cystic duct created by the Endoloop. The choledochoscope most frequently used in our series was the SpyGlass™ Discover (Boston Scientific Corporation, Marlborough, MA, USA), a 3.5 mm (10.5 Fr) instrument with a 65 cm working length, 0° forward view, and 120° field of vision. This device includes two irrigation channels and a 1.2 mm (3.6 Fr) suction/accessory channel, allowing 30° angulation when an accessory device is in place. The video output was connected to one of the laparoscopic monitors, positioned at the patient’s right shoulder. With this setup, transcystic bile duct exploration was initiated (Fig. 2).

**Fig. 2:**
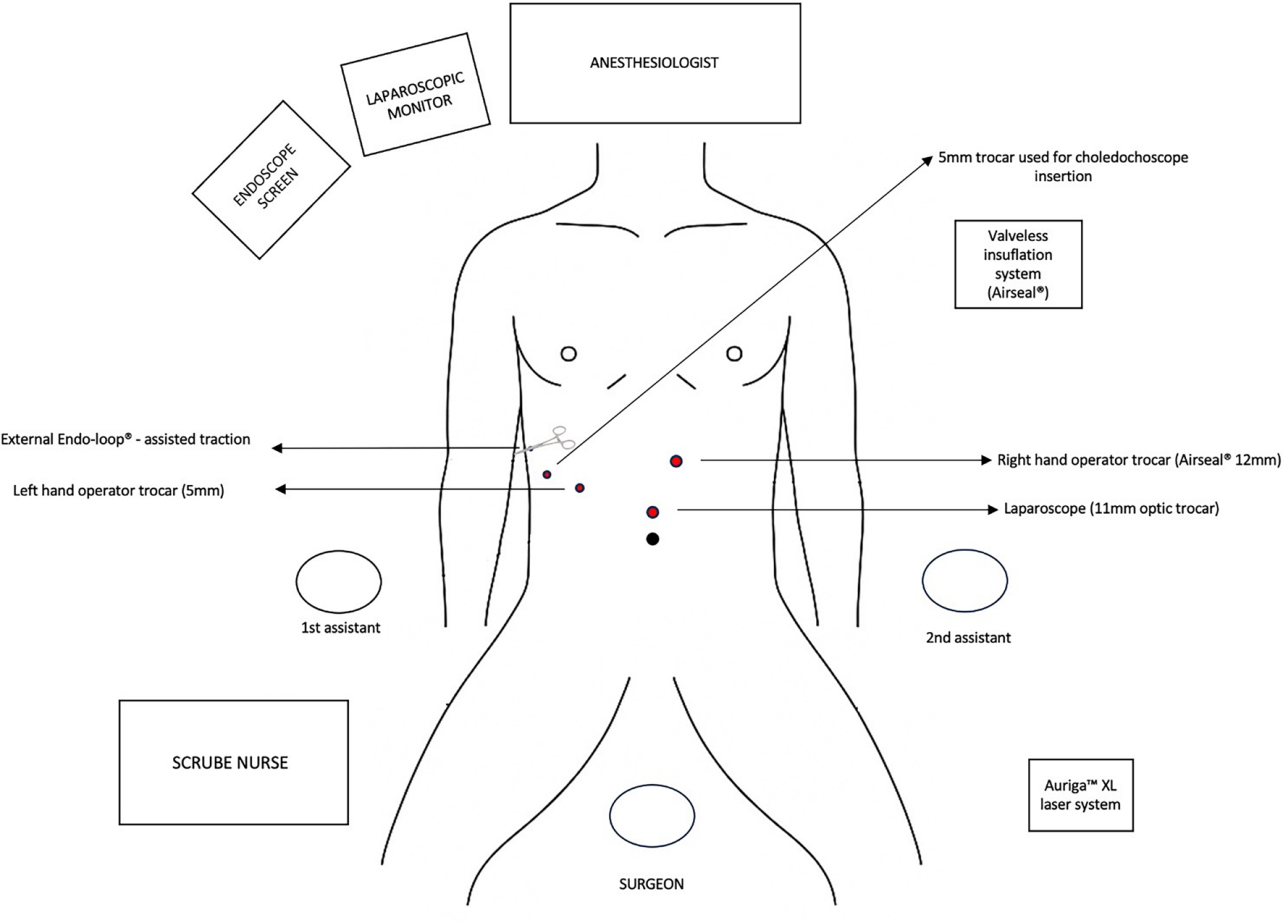
Illustration of the positioning of the patient, surgical team, and instruments used during the procedure. Endoloop-assisted traction maintained externally with a hemostatic clamp is shown
3. During choledochoscopy, the second assistant (standing on the patient’s right side) must maintain upward traction of the liver to facilitate access for the SpyGlass™system. Introduction of the scope into the cystic duct is aided by gentle traction and alignment using a dissector inserted through the right-hand trocar, controlled by the first assistant. Once cannulation of the bile duct with the SpyGlass™ system is achieved, direct choledochoscopy is performed.
4. The exploration begins caudally to the cystic duct, which is anatomically easier to access. Throughout this step, nursing assistance (positioned to the patient’s right) is essential for controlled saline irrigation on demand**,** ensuring adequate visualization of the biliary lumen.
  At this stage, several intraoperative findings may be encountered:
  Clean bile duct: Exploration extends distally to the duodenal papilla (Fig. 3). It is possible to surpass it and perform duodenoscopy. A rectal indomethacin suppository is administered prior to papillary manipulation.

**Fig. 3:**
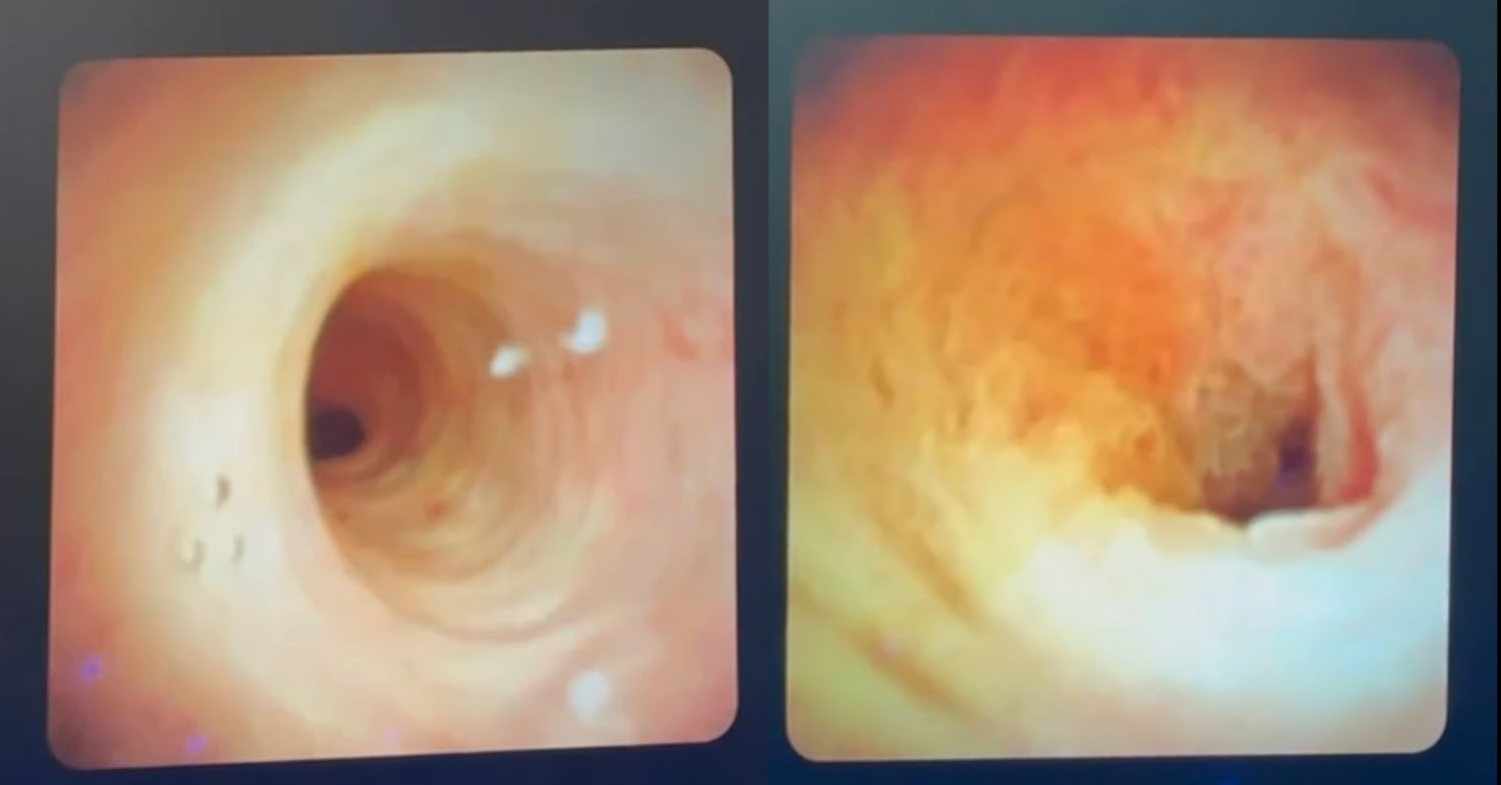
Direct visualization of a clean bile duct during transcystic choledochoscopy. The distal limit of exploration is the duodenal papilla, which can be occasionally traversed to perform duodenoscopy
  Sludge: Mobilized or flushed using pressurized saline irrigation and aspirated. High-flow irrigation through the choledochoscope allows small fragments to pass spontaneously through the papilla. When the cystic duct diameter permits, the Elefant® system (Coloplast, Humlebæk, Denmark) can also be used as a laparoscopic suction–irrigation device**.**
  Mobile stones: When stones are freely mobile, transcystic extraction is attempted using a retrieval basket (Fig. 4). In our series, the NGage® device (Cook Medical) was the most frequently used, followed by the Dormia® N.Stone® (Coloplast). This step may become time-consuming when multiple stones are present. As the primary surgeon operates both hands to manipulate the choledochoscope, assistance from nursing staff or an assistant is required to open and close the retrieval basket. Stones are extracted either through the working channel of the choledochoscope or, alternatively, externalized under direct vision and aspirated or retrieved by the assistant using the surgeon’s now-free right-hand trocar. The correlation between preoperative imaging and the number of extracted stones was not considered essential, given the dynamic nature of this pathology.

**Fig. 4:**
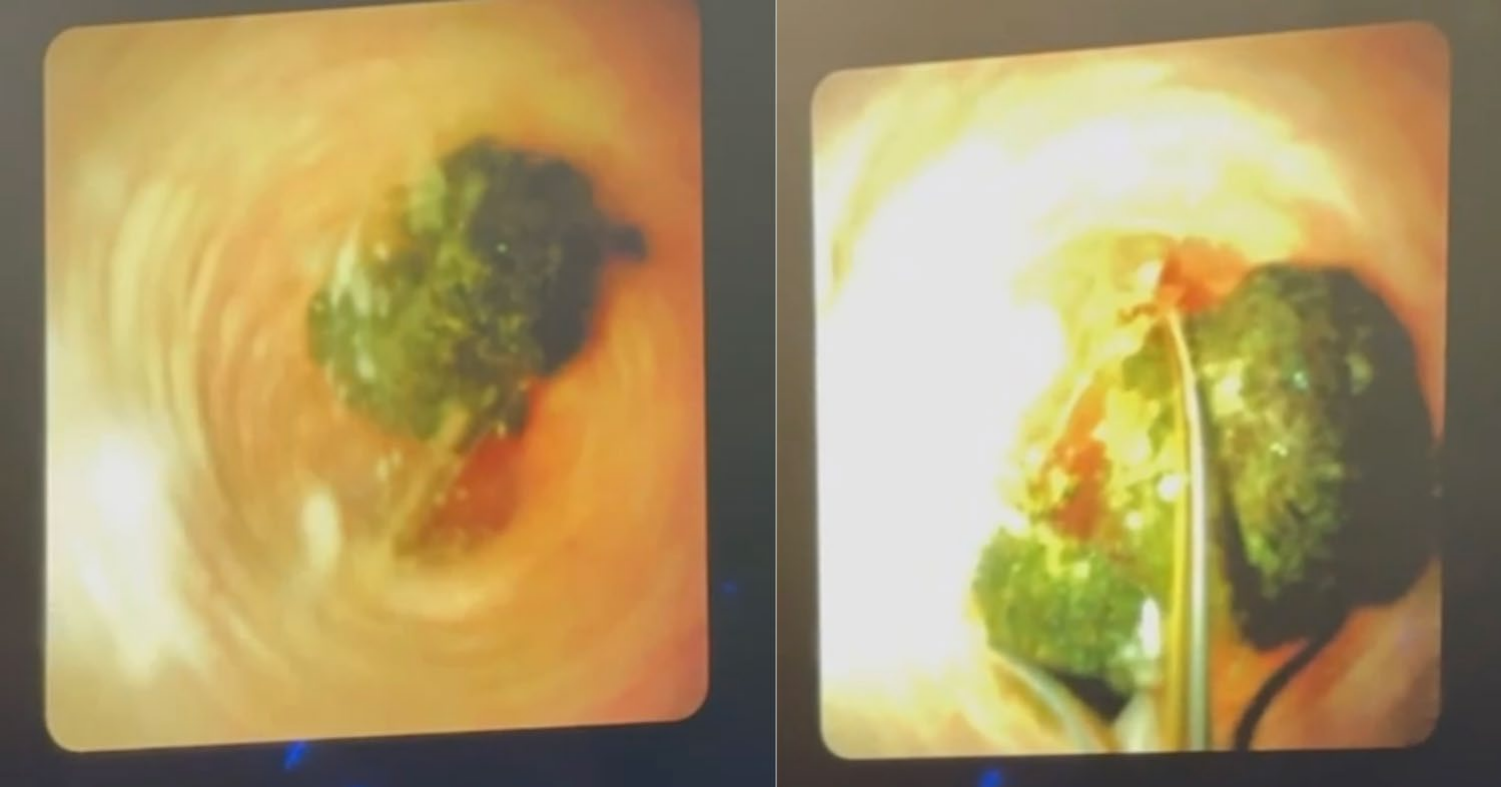
Direct visualization and extraction of a mobile bile duct stone using an NGage® retrieval basket
  Impacted or large stones: For impacted stones or those exceeding the basket size (11–13 mm), laser lithotripsy was performed using the Auriga XL laser system (Boston Scientific Corporation) and a 270 μm Holmium:YAG fiber (Cook Medical). In our series, 46.3% of patients required laser lithotripsy. The fiber tip is placed perpendicular to the center of the stone, and the device is activated under direct vision, taking care to maintain a safe distance from the biliary mucosa (Fig. 5). This technique—combining intermittent laser pulses with simultaneous irrigation—minimizes the risk of iatrogenic injury.

**Fig. 5:**
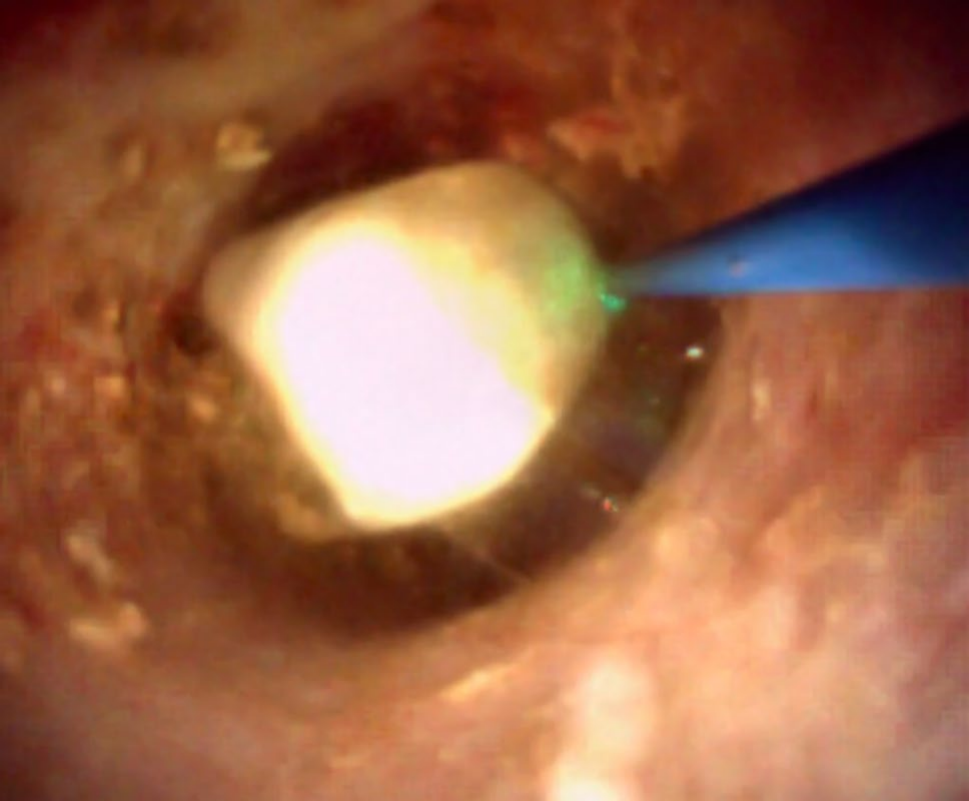
Laser lithotripsy of an impacted bile duct stone using the Auriga XL Holmium:YAG system. The laser fiber is positioned perpendicularly to the stone surface under continuous irrigation to minimize mucosal injury
    We recommend initial settings of 1 joule (J) pulse energy (PE) and 5.0 hertz (Hz) frequency**.** Increasing the PE enhances fragmentation, whereas lower PE promotes stone pulverization. High PE settings are ideal for fragmentation followed by active basket retrieval, while low PE is preferred when performing dusting (powderization) to create fine debris for spontaneous passage [15, 16]. This latter strategy may be particularly useful in patients with failed ERCP who nevertheless underwent successful sphincterotomy.
    When employing high PE, high frequency (Fr) increases stone retropulsion**,** which is why fragmentation and retrieval are typically performed at low Fr settings [12].
    In our series, the mean ± SD energy delivered was 0.978 ± 0.440 J, with a median of 1.2 J [0.5–1.2] and a range of 0.5–1.8 J. The mean frequency applied was 6.2 ± 1.436 Hz, with a median of 5.0 Hz [5.0–8.0] and an overall range of 5.0–8.0 Hz.
    Total power (Power = PE × Fr) should always be considered when selecting parameters for laser lithotripsy, as high power increases heat generation and may result in thermal tissue injury [15]. In experimental porcine models, 40 W applied for 18 s was required to produce significant thermal damage—values rarely reached with our recommended settings.
    Laser lithotripsy typically allows subsequent mobilization and extraction of fragments. However, in some cases—such as very large stones or long casts**—**mechanical lithotripsy (crush maneuver) was employed. This consists of applying external pressure with a laparoscopic grasper, compressing the bile duct and its contents. Through alternating laser and mechanical lithotripsy**,** large or impacted stones are often converted into small, mobile fragments that can be managed using the techniques previously described.
5. Once the distal bile duct has been cleared, we recommend proceeding with cranial exploration toward the cystic duct**.** For this purpose, the choledochoscope is withdrawn to the level of the cystic–common duct junction, and a “windscreen wiper” maneuver, as described by Martínez-Isla and Naratne [17], is performed. This maneuver begins with the choledochoscope oriented distally; controlled anticlockwise torque is then applied using the right thumb and index finger, allowing gentle proximal rotation of the scope. At this point, depending on the intraoperative findings, we proceed according to the specific scenarios described in section 4.
6. Intraoperative cholangiography is not routinely performed and is reserved for cases in which complete clearance of the bile duct is uncertain—particularly if any portion of the exploration, distal or proximal to the cystic duct, could not be satisfactorily completed. For this purpose, an Open-End Flexi-Tip® 5F catheter (Cook Medical) is inserted through the cystic duct and secured with laparoscopic clips**.** Omnipaque™ (iohexol) (GE Healthcare, Oslo, Norway) is used as contrast medium, and a mobile C-arm is employed for Imaging. With appropriate table tilt and the anesthesiologist inducing a short period of apnea, the entire biliary tree should be visualized, with clear passage of contrast into the duodenum (Fig. 6).

**Fig. 6:**
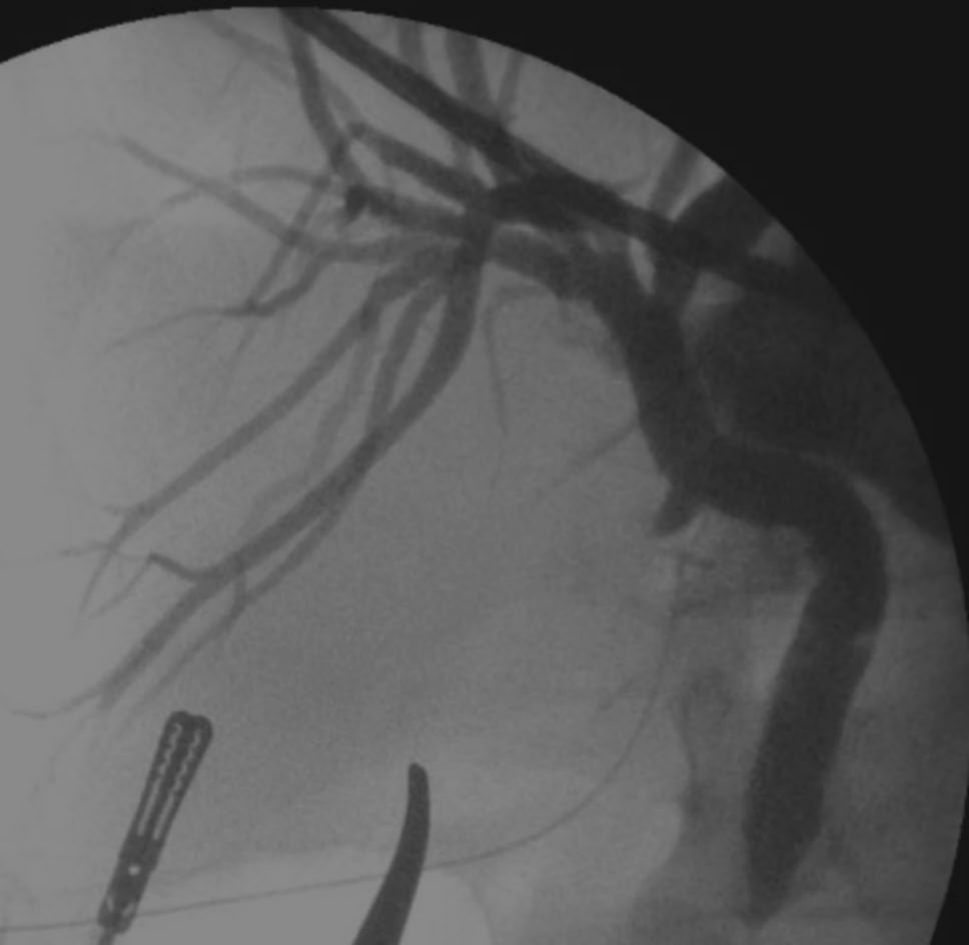
Intraoperative cholangiography performed through the cystic duct using a 5F open-end catheter. Contrast passage to the duodenum confirms complete clearance of the bile duct
7. Once exploration is complete and bile duct clearance has been achieved, the cystic duct stump is closed using two non-absorbable clips or Hem-o-lok® clips. In the few cases where the transcystic approach was not feasible (1% infundibular and 5% choledochotomy in our series), choledochorrhaphy was performed with a continuous 5/0 PDS spiral knotless suture**.** The gallbladder is then extracted within an endoscopic retrieval bag through the supraumbilical trocar.
8. The decision to place an intra-abdominal drain was made according to the intraoperative findings. In our series, a drain was left in 63% of cases; however, it is worth noting that most of these patients correspond to the initial phase of our learning curve. As our experience increased and the technique became more standardized, the trend has progressively shifted toward a more selective and infrequent use of drainage.

### Tips and tricks in different scenarios

**Fig. 7:**
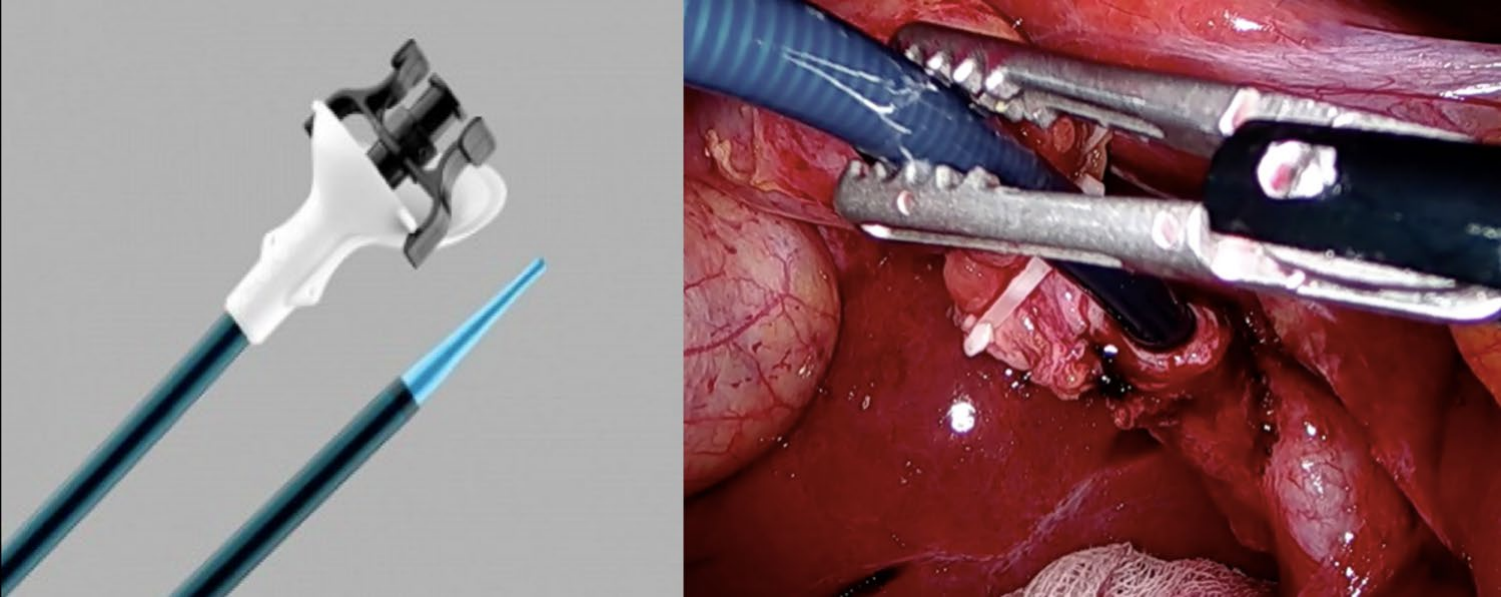
Use of the Flexor® Parallel Access System to facilitate transcystic access in a patient with a narrow cystic duct. This maneuver allows gentle dilation of the cystic duct and disruption of Heister’s valves
  These devices are introduced through the last-placed trocar and are guided intra-abdominally using the surgeon’s right hand. Following the guidewire, they are advanced into the bile duct, thereby achieving cystic duct dilation and disruption of Heister’s valves, which facilitates choledochoscope passage. It is generally unnecessary to maintain the sheath once adequate dilation has been achieved; however, if required, it may remain in place throughout the procedure.
- (B) Transcholedochal Approach Under Specific Conditions
  In certain cases, despite meticulous dissection and prior dilation, a transcystic approach cannot be completed. We have observed this in patients with cystic ducts smaller than 3 mm, low insertions, Mirizzi syndrome, large stones that cannot be extracted through the cystic duct, or in previously cholecystectomized patients. Under these circumstances, a transcholedochal approach is indicated.
  Ideally, access to the common bile duct should be performed as close as possible to the cystic duct stump, which provides a wider opening and improved maneuverability for both cranial exploration and extraction of large stones. The procedure begins with careful identification and dissection of the common bile duct. Two traction sutures are placed proximal and distal to the intended choledochotomy site, allowing controlled mobilization of the duct to facilitate choledochoscope insertion. A mini-longitudinal choledochotomy is then performed on the anterior surface for choledochoscope access.
  Subsequent steps in the exploration are identical to those described previously. Upon completion, the common bile duct is closed either primarily with a 5/0 Stratafix™ suture or with the placement of a T-tube drain, depending on patient characteristics and the pathology encountered.
- (C) Transinfundibular Approach/Cystic Rupture (Detached Gallbladder)
  In certain situations—particularly in cases of severe inflammatory pathology or advanced cholecystitis, where cystic duct dissection becomes impossible—a transinfundibular approach is preferred. A traction suture is placed in the infundibulum and externalized percutaneously through the right hypochondrium using an EndoClose® device.
  An incision is then made in the lower portion of the infundibulum, and the exit of the cystic duct is gently cannulated using a dissector or similar instrument to enable choledochoscope insertion. Once the cystic duct is identified, if necessary, the dilation maneuvers described in Section A may be applied to widen the access and facilitate instrument passage.
  This approach is also applicable in cases where, during traction maneuvers, the cystic duct tears and the gallbladder becomes detached. A traction suture is placed at the most distal end of the cystic duct stump, and a new proximal access is created (Fig. 8).

- (A) Narrow Cystic Duct
  In our series, 67% of patients did not require the use of any introducer; however, in cases of narrow, fibrotic, or retracted cystic ducts, we have resorted to using dilators or access sheaths to facilitate the introduction of the choledochoscope. In our series, the most frequently used device was the Flexor® Parallel Access System, 12 Fr (Cook Medical, Bloomington, IN, USA) (Fig. 7). In other cases, a urological dilator (6–18 Fr, gradual dilation) was employed, guided by the SpyGlass™ Discover Jagwire GuideWire (Boston Scientific, Marlborough, MA, USA). On rare occasions, a Fogarty catheter was used.

**Fig. 8:**
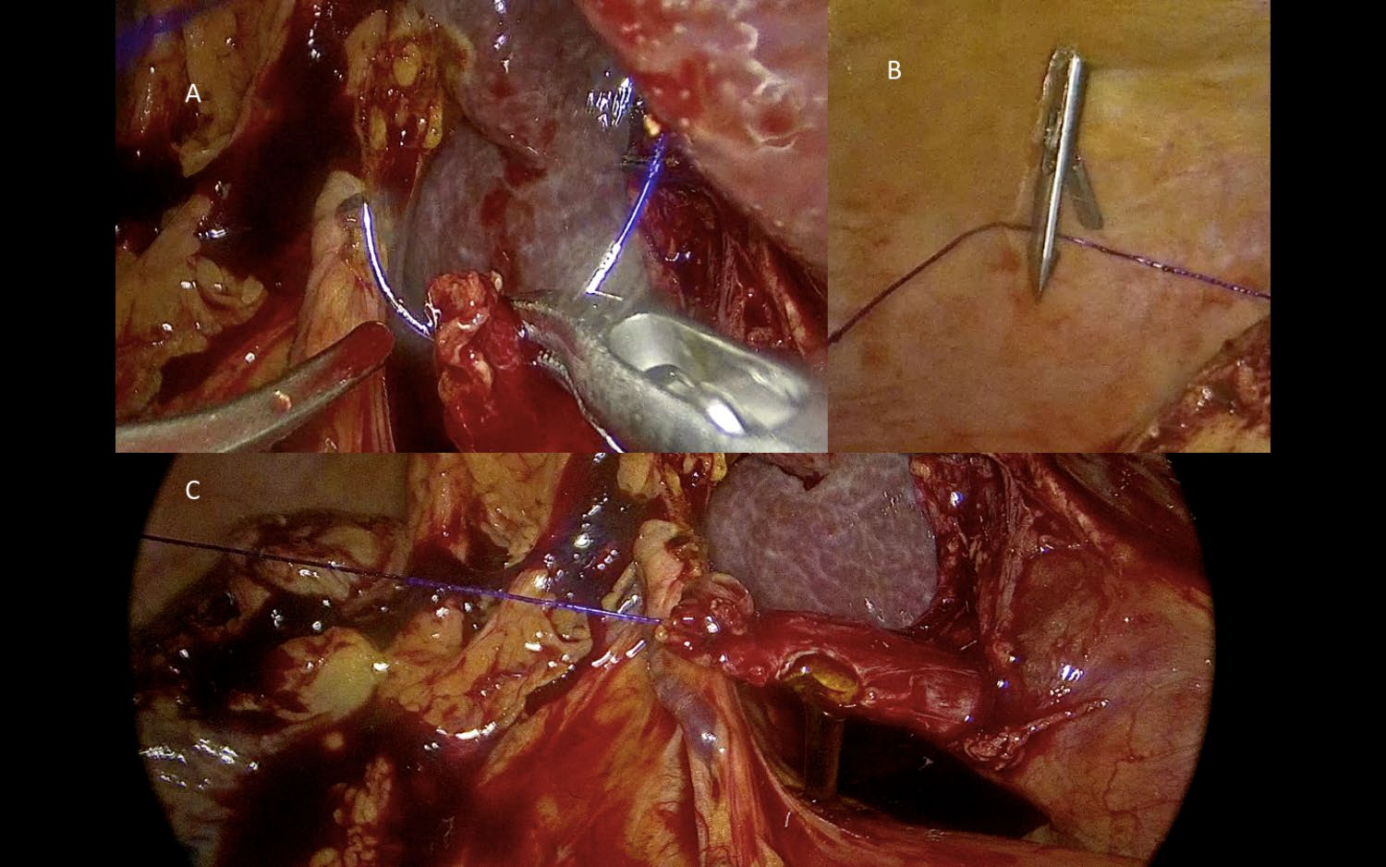
Management of cystic duct avulsion during traction maneuvers. A new traction suture is placed at the most distal end of the cystic duct stump to establish a new proximal access for choledochoscopy
- (D) Presence of a Biliary Stent
  When a pre-existing biliary stent is present and has not been removed, the transcystic approach should not be disregarded, regardless of the stent’s height or its relationship to the cystic duct junction. In these cases, it is crucial to perform the transcystic access as close as possible to its confluence with the common bile duct to allow stent removal.
  After the cystic duct is incised, the choledochoscope is gently introduced. Using the fiber of the laser system, we release the adhesions between the stent and the bile duct wall under direct visualization. Once adequate mobility of the stent is confirmed, it is grasped blindly with a laparoscopic grasper and extracted from the bile duct. Routine bile duct exploration is then continued as described above.

Postoperative outcomes demonstrated a successful common bile duct (CBD) stone clearance rate of 98.95% (95/100 patients). Clearance was not achieved in one patient (1.04%), and other findings, including tumoral dilatation, were reported in four cases.

Overall morbidity, according to the Clavien–Dindo classification, was distributed as follows: 67 patients (67%) experienced no complications (grade 0), 8 (8%) had grade I complications, 14 (14%) grade II, 7 (7%) grade IIIa, 2 (2%) grade IIIb, and 2 patients (2%) died (grade V). No grade IV complications were observed.

Bile leak occurred in 9% of patients. According to the International Study Group of Liver Surgery (ISGLS) criteria [18], bile leaks were graded as type A in 2 patients (22.22%), type B in 3 (33.33%), and type C in 4 (44.44%). Postoperative bleeding was reported in 2 patients (2%), pancreatitis in 3 (3%), and CBD stricture in 3 (3%).

The mean length of hospital stay was 4.86 ± 3.75 days (median 4.00; interquartile range [IQR] 3.00–6.00; range 1.00–21.00). Laboratory parameters obtained one month after surgery showed a mean total bilirubin level of 0.81 ± 0.64 mg/dL (median 0.80; IQR 0.45–1.00; range 0.17–5.39), gamma-glutamyl transferase (GGT) of 100.69 ± 121.57 U/L (median 69.00; IQR 20.00–108.00; range 8.00–692.00), and alkaline phosphatase (ALP) of 112.83 ± 56.14 U/L (median 99.00; IQR 76.00–141.00; range 40.00–374.00).

Readmission occurred in 11 patients (11%). The causes of readmission were missed stones in 3 cases (27.27%), bile leak in 2 (18.18%), cholangitis in 1 (9.09%), prosthesis removal in 1 (9.09%), postoperative collection in 1 (9.09%), and other causes in 3 patients (27.27%). Reoperation was required in 4 patients (4%), due to malignant pathology in 2 cases (50%), bile leak in 1 (25%), and bleeding in 1 (25%). The 90 day mortality rate was 2%.

**Fig. 9:**
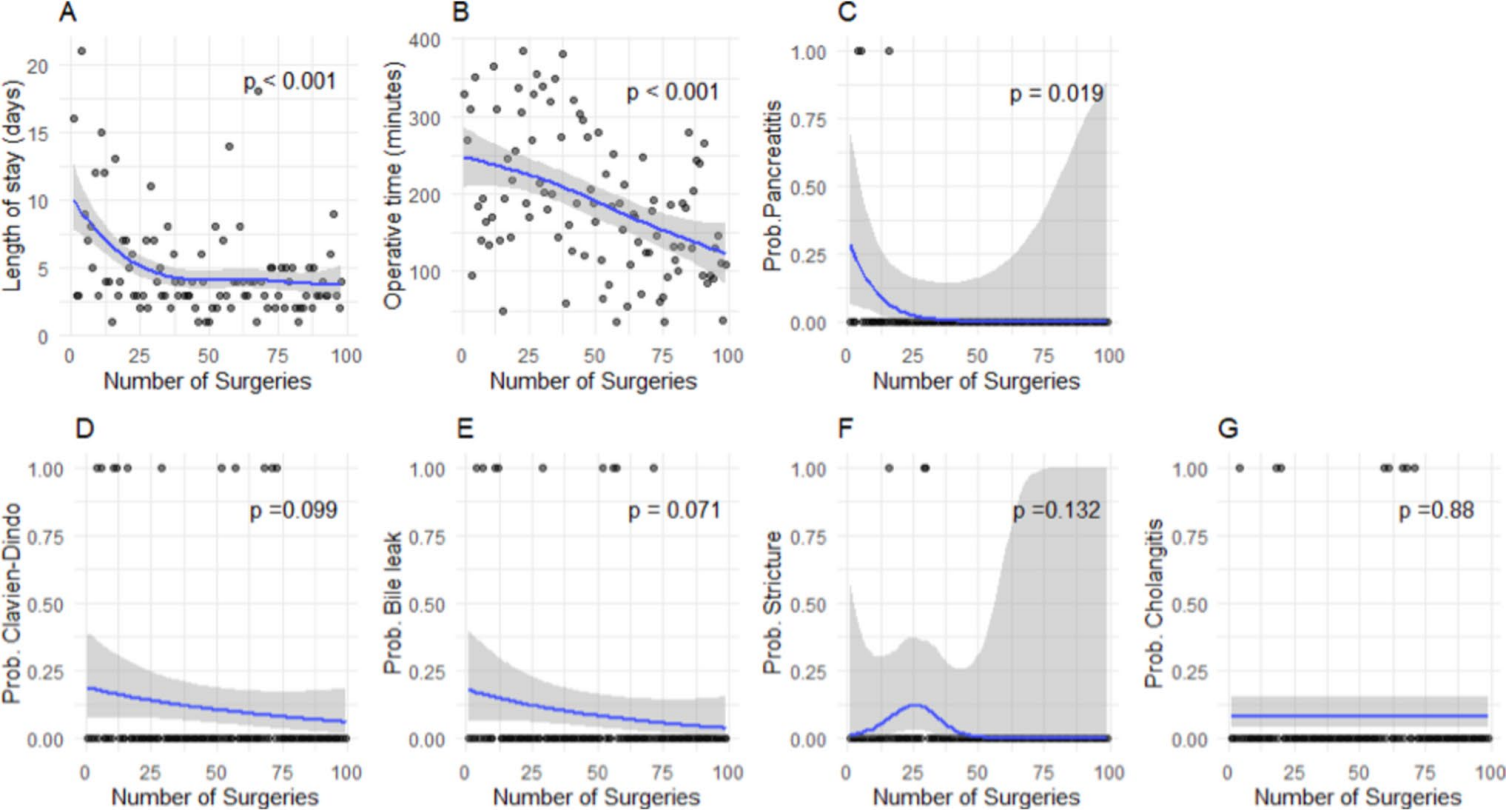
Univariate effect of cumulative procedure number on postoperative outcomes (GAM model). The solid line represents the estimated mean, and the shaded band represents the 95% confidence interval

Figure 9 shows the univariate effect of cumulative procedure number on postoperative outcomes. Analysis of the learning curve demonstrated a significant non-linear association between increasing procedural volume and improvements in early outcomes, particularly operative time, postoperative length of stay and the incidence of postoperative pancreatitis. Most adverse events clustered within the initial phase of the series, with a clear reduction observed after a defined number of accumulated cases, but the relationship with the other variables was not statistically significant.

Table 1 evaluates the effect of the learning curve. The relationship between cumulative case number and the first three outcomes appears to be nonlinear and significant; however, the relationship with the remaining outcomes was not significant (it cannot be predicted whether the probability of complications, leaks, strictures, or cholangitis increases or decreases as the surgeon gains experience). In both the first three outcomes and the subsequent ones, other variables were statistically significant (*p* < 0.05) in the adjusted models, meaning they do predict the occurrence of specific outcomes: age, preoperative leukocyte count, and stone size (predict length of stay); bile duct diameter, stone size, and CCI (predict operative time); bile duct diameter (predicts pancreatitis); bile duct diameter (predicts cholangitis); age (predicts leaks); no variable predicted strictures; and age and leukocyte count (predict complications). Importantly, this modeling approach not only characterizes the learning curve but also provides quantitative estimates of the number of cases required to achieve acceptable outcome benchmarks in centers adopting this technique, thereby offering practical guidance on how much experience is needed to reach stable and satisfactory performance.

**Table 1:** Generalized additive models (GAM) and evaluation the effect of the learning curve

Outcomes	Median [IQR]/n (%)	Model	GAM assumised distribution	Effective degrees of freedom (edf)	p*	R^2^ adj total	Criterion	Number of surgery cases estimated	Figure 9
Length of stay^b^ (days)	4 [3–5.5]	Uni	Poisson	2.865	** < 0.001**	0.177			A
		Adj		2.936	** < 0.001**	0.327	< 5 days	29	
Operative time (minutes)	183 [125.3–253.5]	Uni	Gaussian	1.547	** < 0.001**	0.197			B
		Adj		3.053	** < 0.001**	0.441	< 160 min	50	
Pancreatitis	3 (3.03%)	Uni	Binomial	0.833	**0.019**	0.129			C
		Adj		0.800	**0.025**	0.375	< 1%	32	
Clavien–Dindo > / = 11	11 (11.11%)	Uni	Binomial	0.652	0.099	0.020			D
		Adj		0.522	0.16	0.129			
Bile leak	9 (9.09%)	Uni	Binomial	0.760	0.071	0.042			E
		Adj		0.691	0.095	0.131			
Stricture	3 (3.03%)	Uni	Binomial	0.627	0.132	0.012			F
		Adj		0.566	0.136	0.018			
Cholangitis	8 (8.08%)	Uni	Binomial	0.001	0.688	0.001			G

Bold values indicate statistical significance in the adjusted models

*GAM* generalized additive model

*The impact of cumulative procedure number on each outcome was assessed unadjusted (Uni) and adjusted (Adj) for age, preoperative leukocyte count, bile duct diameter, stone size, and Charlson comorbidity index (CCI). Penalized cubic regression splines with 10 degrees of freedom were used to capture potential nonlinear relationships while limiting model complexity to avoid overfitting

Examples of interpretation for the criterion column: “For a patient with median values of age, leukocytes, bile duct diameter, stone size, and CCI, the model predicts a mean length of stay of 5 days when the cumulative number of surgeries reaches approximately 29 cases”; “For a patient with median values of age, leukocytes, bile duct diameter, stone size, and CCI, the model predicts a probability of less than 1% of developing pancreatitis when the cumulative number of surgeries reaches approximately 32 cases

## Discussion

As previously mentioned, we consider tLCBDE the first-line approach for managing patients with choledocholithiasis and gallbladder in situ, regardless of cystic duct diameter, stone burden or size, and patient characteristics [12–14].

The implementation of this surgical technique naturally requires specific training, as the learning curve significantly influences both outcomes and success rates [19, 20]. tLCBDE reduces morbidity compared to the transcholedochal approach, lowers the risk of bile leakage and stricture formation, and shortens hospital stay [5, 9, 10]. Reported success rates in the literature exceed 85–90% in experienced centers [12]. According to published data, the learning curve for tLCBDE ranges from approximately 60–80 cases to achieve competence and results comparable to those of expert surgeons [21]. Several studies and guideline reviews including the SAGES highlight the importance of structured training and institutional support for LCBDE implementation [5].

Analyses using various models describe the curve as consisting of distinct phases—initial learning, competence, and mastery—with progressive reductions in operative time and complications as experience increases [22]. Structured learning, including mentorship programmes and simulation-based training, could effectively shorten the learning curve and facilitate successful adoption of tLCBDE [23, 24]. Nevertheless, several factors influence the duration of this curve. “Easier” cases—such as those with a wide cystic duct, single and small stones, absence of acute inflammation, or normal anatomy—facilitate faster progression. However, as in our series, when the technique is implemented by surgeons already highly experienced in advanced laparoscopic procedures and biliary surgery, the learning curve tends to be shorter, enabling competence in more complex scenarios after fewer cases [25]. These include the management of large or multiple stones, anatomical variations, narrow cystic ducts, emergency situations, and patients with significant comorbidities.

The learning curve for tLCBDE primarily affects efficiency-related outcomes and selected early postoperative complications, while more complex biliary events appear to be influenced predominantly by patient and disease specific factors rather than surgical experience alone. The concentration of adverse events in the initial cases underscores the importance of structured training, careful patient selection during early implementation, and team standardization. Overall, our results support the notion that acceptable and stable outcomes can be achieved after a relatively limited number of cases, reinforcing the feasibility of tLCBDE adoption in appropriately prepared centers.

Cost-effectiveness also plays a role in the adoption of this technique. Multiple economic analyses have shown that single-stage strategies—such as tLCBDE or intraoperative ERCP—tend to be more cost-effective than two-stage approaches involving preoperative ERCP [26, 27]. The comparative costs between tLCBDE and intraoperative ERCP are influenced by local surgical expertise and available resources; however, centers with well-established laparoscopic programs frequently report lower overall expenditures with the transcystic approach. Accordingly, in our current institutional protocol, transcystic bile duct exploration is considered the preferred first-line intervention for patients with choledocholithiasis and an intact gallbladder, regardless of intraoperative findings or patient-specific considerations.

Conducting this study in a regional hospital with a large and unselected cohort enhances its external validity and demonstrates that favourable outcomes with tLCBDE can be achieved in settings similar to ours. In our study, the CBD clearance rate was 95%, which is comparable to previously published outcomes in both randomized [28, 29] and non-randomized studies [6, 30, 31] where clearance rates ranged from 71 to 98%. The proportion of patients in the tLCBDE group who experienced a postoperative complication of Clavien–Dindo grade ≥ III was 11%. Although this figure is slightly higher than the 2.8–7.1% reported in other series [30, 32–34], it is important to note that most of these complications occurred during the early phase of the learning curve. The median length of hospital stay in our cohort was 4 days, which is similar the median ranges of 3–5 days described elsewhere [6, 30].

The readmission rate was high at 11%, compared with previously reported rates of 0–8.9% [13, 32, 33, 35]. However, this difference may be partly explained by the fact that our analysis included all readmissions, irrespective of whether they were directly related to the procedure.

Our higher bile-leak rate may be partly attributable to the characteristics of our cohort, which included a larger proportion of patients with previous biliary interventions—mainly ERCP—known to increase local inflammation and technical difficulty. We also treated more patients with higher ASA scores, reflecting greater systemic comorbidity. Nevertheless, these factors did not prolong operative time, with a median duration of 189 min, comparable to other series: 164–191 min [12, 35]. Importantly, the learning-curve analysis shows a clear downward trend in operative time and bile-leak incidence as experience increased, suggesting that early-phase cases disproportionately influenced the overall rate.

Early postoperative biliary strictures were observed in 3 patients (3%), a slightly higher rate than in other series. Despite loss to follow-up in one case, no persistent abnormalities were seen on MRCP at 6 months, suggesting that these strictures were transient inflammatory changes rather than true established lesions [14].

tCBDE has, in recent years, transformed the paradigm of choledocholithiasis management, with an increasing number of clinical guidelines supporting it as the preferred first-line approach in experienced centers. We believe that the standardization of this procedure is a critical step toward validating the technique as a robust therapeutic alternative to any other form of treatment. It is precisely through technical standardization that our center has been able to offer this approach as the primary therapeutic option, regardless of the particularities of the disease or the patient.

## Conclusion

The transcystic approach appears to be a valid, reproducible, and safe first-line technique for the management of patients with choledocholithiasis and gallbladder in situ—regardless of the number or size of the stones, the diameter of the cystic or common bile duct, the clinical or biochemical presentation, or patient-specific characteristics.

Our experience supports its feasibility across a wide spectrum of scenarios, including complex cases traditionally considered unsuitable for transcystic management. The progressive refinement and standardization of the procedure have proven essential not only for ensuring consistent outcomes but also for shortening the learning curve, allowing surgeons to achieve proficiency more rapidly and with lower complication rates.

## Supplementary Information

Below is the link to the electronic supplementary material.

Supplementary file1 (M4V 27739 KB)

## References

[1] Ramia JM, Parra-Membrives P (2021) Cirugía de la litiasis biliar. In: Parrilla Paricio P (ed) Cirugía AEC, 3rd edn. Editorial Médica Panamericana, Madrid, pp 819–827

[2] Marks B, Al Samaraee A (2021) Laparoscopic exploration of the common bile duct: a systematic review of the published evidence over the last 10 years. Am Surg 87(3):404–418. 10.1177/0003134820949527 33022185 10.1177/0003134820949527

[3] Ricci C, Pagano N, Taffurelli G et al (2018) Comparison of efficacy and safety of 4 combinations of laparoscopic and intraoperative techniques for management of gallstone disease with biliary duct calculi: a systematic review and network meta-analysis. JAMA Surg 153:e181167 29847616 10.1001/jamasurg.2018.1167 PMC6137518

[4] Prasson P, Bai X, Zhang Q, Liang T (2016) One-stage laparoendoscopic procedure versus two-stage procedure in the management for gallstones disease and biliary duct calculi: a systematic review and meta-analysis. Surg Endosc 30(8):3585–3590 10.1007/s00464-015-4657-0 26718360

[5] Zerey M, Haggerty S, Richardson W et al (2018) Laparoscopic common bile duct exploration. Surg Endosc 32:2603–2612. 10.1007/s00464-017-5991-1 29273878 10.1007/s00464-017-5991-1

[6] Wahi JE, Warmack T, Barghout R et al (2023) Five-year experience with transcystic laparoscopic common bile duct exploration. J Laparoendosc Adv Surg Tech A 33(3):276–280. 10.1089/lap.2022.0408 36459625 10.1089/lap.2022.0408

[7] Zhu H, Xu M, Shen H, Yang C, Li F, Li K et al (2015) A meta-analysis of single-stage versus two-stage management for concomitant gallstones and common bile duct stones. Clin Res Hepatol Gastroenterol 39(5):584–593 25936687 10.1016/j.clinre.2015.02.002

[8] Feng Q, Huang Y, Wang K, Yuan R, Xiong X, Wu L (2016) Laparoscopic transcystic common bile duct exploration: advantages over laparoscopic choledochotomy. PLoS ONE 11(9):e0162885. 10.1371/journal.pone.0162885 27668730 10.1371/journal.pone.0162885 PMC5036868

[9] Hajibandeh S, Hajibandeh S, Sarma DR et al (2019) Laparoscopic transcystic versus transductal common bile duct exploration: a systematic review and meta-analysis. World J Surg 43(8):1935–1948. 10.1007/s00268-019-05005-y 30993390 10.1007/s00268-019-05005-y

[10] Pang L, Zhang Y, Wang Y, Kong J (2018) Transcystic versus traditional laparoscopic common bile duct exploration: its advantages and a meta-analysis. Surg Endosc 32(11):4363–4376. 10.1007/s00464-018-6286-x 29943056 10.1007/s00464-018-6286-x

[11] Navaratne L, Martínez-Isla A (2021) Transductal versus transcystic laparoscopic common bile duct exploration: an institutional review of over four hundred cases. Surg Endosc 35(1):437–448. 10.1007/s00464-020-07522-7 32246237 10.1007/s00464-020-07522-7

[12] Huang J, Hu W, Liu J et al (2023) Laparoscopic transcystic common bile duct exploration: 8-year experience at a single institution. J Gastrointest Surg 27(3):555–564. 10.1007/s11605-023-05594-z 36652180 10.1007/s11605-023-05594-z

[13] Berg LS, Friis-Andersen H, Zinther NB, Öztoprak M, Gotschalck KA (2025) Feasibility and outcome of transcystic laparoscopic common bile duct exploration as first-line treatment for common bile duct stones: a retrospective cross-sectional study. Surg Endosc 39(4):2256–2266. 10.1007/s00464-025-11587-7 39934279 10.1007/s00464-025-11587-7

[14] Barros VN, Murillo LA, Vozmediano RC, Vásquez CG, Marchán SJ (2025) Transcystic management of choledocholithiasis: outcomes, factors associated with bile duct injury and implications for surgical practice. Ann Hepatobiliary Pancreat Surg. 10.14701/ahbps.25-145 41157820 10.14701/ahbps.25-145 PMC12643810

[15] Black KM, Aldoukhi AH, Ghani KR (2019) A users guide to Holmium laser lithotripsy settings in the modern era. Front Surg 6:48. 10.3389/fsurg.2019.00048 31475152 10.3389/fsurg.2019.00048 PMC6702256

[16] Sea J, Jonat LM, Chew BH, Qiu J, Wang B, Hoopman J et al (2012) Optimal power settings for Holmium:YAG lithotripsy. J Urol 187:914–919. 10.1016/j.juro.2011.10.147 22264464 10.1016/j.juro.2011.10.147

[17] Martinez-Isla A, Navaratne L (eds) (2022) Laparoscopic common bile duct exploration. Springer, Cham. 10.1007/978-3-030-93203-9

[18] Koch M, Garden OJ, Padbury R et al (2011) Bile leakage after hepatobiliary and pancreatic surgery: a definition and grading of severity by the International Study Group of Liver Surgery. Surgery 149(5):680–688. 10.1016/j.surg.2010.12.002 21316725 10.1016/j.surg.2010.12.002

[19] Zhu JG, Han W, Guo W, Su W, Bai ZG, Zhang ZT (2015) Learning curve and outcome of laparoscopic transcystic common bile duct exploration for choledocholithiasis. Br J Surg 102(13):1691–1697. 10.1002/bjs.9922 26395452 10.1002/bjs.9922

[20] Chuang SH, Hung MC, Huang SW, Chou DA, Wu HS (2018) Single-incision laparoscopic common bile duct exploration in 101 consecutive patients: choledochotomy, transcystic, and transfitulous approaches. Surg Endosc 32(1):485–497. 10.1007/s00464-017-5658-y 28643057 10.1007/s00464-017-5658-y

[21] Durán M, Silvestre J, Hernández J, Briceño J, Martínez-Isla A, Martínez-Cecilia D (2023) Learning curve for performing laparoscopic common bile duct exploration in biliary surgery 2.0 era. J Hepatobiliary Pancreat Sci 30(3):374–382. 10.1002/jhbp.1228 35947065 10.1002/jhbp.1228

[22] Chan KS, Teo ZHT, Oo AM, Junnarkar SP, Shelat VG (2023) Learning curve of laparoscopic common bile duct exploration: a systematic review. J Laparoendosc Adv Surg Tech A 33(3):241–252. 10.1089/lap.2022.0382 36161969 10.1089/lap.2022.0382

[23] VanDruff VN, Santos BF, Kuchta K, Cotter R, Goldwag J, Cai M, Fowler X, Lamb CR, Uyrga AJ, Cutshall M, Davis BR, Lerma RA, Auyang ED, Li W, Ceppa EP, Jones E, Abbitt D, Amundson JR, Joseph S, Hedberg HM, McCormack M, Ujiki MB (2024) The laparoscopy in biliary exploration research and training initiative (LIBERTI) trial: simulator-based training for laparoscopic management of choledocholithiasis. Surg Endosc 38(2):931–941. 10.1007/s00464-023-10480-5 37910247 10.1007/s00464-023-10480-5

[24] Durán M, Martínez-Cecilia D, Navaratne L, Brice.o J, Martínez-Isla A (2024) Structured learning and mentoring: shortening the learning curve in laparoscopic common bile duct exploration. Surg Endosc 38(12):7172–7178. 10.1007/s00464-024-11304-w 39363103 10.1007/s00464-024-11304-w

[25] Nassar AHM, Sallam M, Khan KS, Kilpatrick R, Zino S, Katbeh TZ (2023) A proposed difficulty grading system for laparoscopic bile duct exploration: benefits to clinical practice, training and research. Surg Endosc 37(9):7012–7023. 10.1007/s00464-023-10169-9 37349591 10.1007/s00464-023-10169-9 PMC10462500

[26] Morrell DJ, Pauli EM, Hollenbeak CS (2022) Inpatient choledocholithiasis management: a cost-effectiveness analysis of management algorithms. J Gastrointest Surg 26(4):837–848. 10.1007/s11605-022-05249-5 35083722 10.1007/s11605-022-05249-5

[27] Ramser B, Coleoglou Centeno A, Ferre A, Thomas S, Brooke M, Pieracci F, Morton A (2024) Laparoscopic common bile duct exploration is an effective, safe, and less-costly method of treating choledocholithiasis. Surg Endosc 38(10):6076–6082. 10.1007/s00464-024-11139-5 39138682 10.1007/s00464-024-11139-5

[28] Barreras Gonz.lez JE, Torres Pe.a R, Ruiz Torres J, Mart.nez Alfonso M, Brizuela Quintanilla R, Morera P.rez M (2016) Endoscopic versus laparoscopic treatment for choledocholithiasis: a prospective randomized controlled trial. Endosc Int Open 04(11):E1188–E1193 10.1055/s-0042-116144 PMC5111834 27857966

[29] Grubnik VV, Tkachenko AI, Ilyashenko VV, Vorotyntseva KO (2012) Laparoscopic common bile duct exploration versus open surgery: comparative prospective randomized trial. Surg Endosc 26(8):2165–2171. 10.1007/s00464-012-2194-7 22350244 10.1007/s00464-012-2194-7

[30] Quaresima S, Balla A, Guerrieri M, Campagnacci R, Lezoche E, Paganini AM (2017) A 23 year experience with laparoscopic common bile duct exploration. HPB 19(1):29–35. 10.1016/j.hpb.2016.10.011 27890483 10.1016/j.hpb.2016.10.011

[31] Estell.s Vidagany N, Domingo Del Pozo C, Peris Tom.s N, D.ez Ares J, V.zquez Tarrag.n A, Blanes Masson F (2016) Eleven years of primary closure of common bile duct after choledochotomy for choledocholithiasis. Surg Endosc 30(5):1975–1982. 10.1007/s00464-015-4424-2 26201414 10.1007/s00464-015-4424-2

[32] ElGeidie AA, ElShobary MM, Naeem YM (2011) Laparoscopic exploration versus intraoperative endoscopic sphincterotomy for common bile duct stones: a prospective randomized trial. Dig Surg 28(5–6):424–431. 10.1159/000331470 22236538 10.1159/000331470

[33] Al-Temimi MH, Kim EG, Chandrasekaran B, Franz V, Trujillo CN, Mousa A, Tessier DJ, Johna SD, Santos DA (2017) Laparoscopic common bile duct exploration versus endoscopic retrogradecholangiopancreatography for choledocholithiasis found at time of laparoscopic cholecystectomy: analysis of a large integrated health care system database. Am J Surg 214(6):1075–1079. 10.1016/j.amjsurg.2017.08.030 28939251 10.1016/j.amjsurg.2017.08.030

[34] Paganini AM, Guerrieri M, Sarnari J, De Sanctis A, D’Ambrosio G, Lezoche G, Perretta S, Lezoche E (2007) Thirteen years’ experience with laparoscopic transcystic common bile duct exploration for stones. Effectiveness and long-term results. Surg Endosc 21(1):34–40. 10.1007/s00464-005-0286-3 17111284 10.1007/s00464-005-0286-3

[35] Al-Ardah M, Barnett RE, Morris S et al (2021) Lessons learnt from the first 200 unselected consecutive cases of laparoscopic exploration of common bile duct stones at a district general hospital. Surg Endosc 35:6268–6277. 10.1007/s00464-020-08127-w 33140155 10.1007/s00464-020-08127-w

